# An environmental risk assessment of maize containing event, DP-Ø51291–2, with activity against corn rootworms (*Diabrotica spp*.) via expression of the protein, IPD072Aa

**DOI:** 10.1080/21645698.2025.2572192

**Published:** 2025-10-22

**Authors:** Kursten A. Spegar, Bridget F. O’Neill, Veríssimo Sá, Kristine LeRoy, Emily Moellring, Sarah Brustkern, Reba Bruyere, Taylor Olson, Tim Gunderson, Philip Utley, Anne B. Carlson, Rachel Woods, Brian Stolte, Chris Linderblood, Nick Schmidt

**Affiliations:** a Corteva Agriscience™, Regulatory and Stewardship, Indianapolis, Indiana, USA; b Corteva Agriscience™, Regulatory and Stewardship, Johnston, Iowa, USA

**Keywords:** Genetically modified, IPD072Aa protein, non-target organism, risk assessment, *Zea mays* L

## Abstract

Maize event DP-Ø51291–2 expresses the protein, IPD072Aa, which is derived from *Pseudomonas chlororaphis*, encoded by the *ipd072Aa gene*, to provide corn rootworm control. An environmental risk assessment was conducted for DP-Ø51291–2 maize which characterized potential exposure and hazard of the IPD072Aa protein to non-target organisms (NTOs). To estimate potential exposure to the IPD072Aa protein, worst-case estimated environmental concentrations (EECs) and refined EECs, where applicable, were calculated. To characterize potential hazard from the IPD072Aa protein, laboratory dietary toxicity studies were conducted with surrogate NTOs representing functional groups selected via problem formulation. Margins of exposure for each surrogate species were determined by comparing hazard and exposure values which indicated negligible potential risk to NTO populations. To add an additional line of evidence, a field assessment was conducted for DP-Ø51291–2 maize as compared to a near isoline control. Overall, no unreasonable adverse effects to NTO populations are anticipated from DP-Ø51291–2 maize cultivation.

## Introduction

According to the United States Department of Agriculture, Foreign Agricultural Service (USDA-FAS), the global production of maize (*Zea mays* L.) from 2023 to 2024 was approximately 1.23 billion metric tons, a six percent increase year over year from previous years. The United States is the world’s largest producer and accounts for 32% (389.69 million metric tons) of global production.^1^ Each growing season presents a wide array of challenges for growers such as disease, weather events, weed pressure, and insect pests. Corn rootworms (*Diabrotica spp*.) are a major pest of maize in Europe, Eastern Canada, and the United States. Within the United States, western corn rootworm (*Diabrotica virgifera virgifera* LeConte) larvae feed on maize roots^2,3^ leading to root injury^4^ that is coupled with decreased plant growth^5^ and yield.^6^ To help combat western corn rootworms, growers typically use one or more of the following management practices: crop rotation; soil/seed/or foliar application of chemicals; and/or genetically modified (GM) maize designed with RNA interference (RNAi) technology^7,8^ and/or to express insecticidal proteins.^9–11^ Over the past two decades, these GM lines have provided options for successful management of western corn rootworm pressure, however, instances of populations resistant to proteins derived from *Bacillus thuringiensis* (*Bt*) expressed in plants are becoming more common.^12–14^ Western corn rootworms have also demonstrated resistance to other pest management options such as pesticides^15^ and crop rotation^16^ indicating the need for proteins with new modes of action against western corn rootworm.

To provide a new insect resistance management option for growers and keep up with the global demand for maize, the GM maize event, DP- Ø51291–2 (hereafter referred to as DP51291 maize) was developed, expressing the insecticidal protein IPD072Aa. The IPD072Aa protein is encoded by the *ipd072Aa* gene, derived from the gram-negative soil bacteria, *Pseudomonas chlororaphis*, and provides protection against certain susceptible corn rootworm (*Diabrotica* spp.) when expressed in plants by causing disruption of the midgut epithelium.^17^ The event also expresses the phosphinothricin acetyltransferase (PAT) protein derived from *Streptomyces viridochromogenes* for tolerance to glufosinate-ammonium, the active ingredient in phosphinothricin herbicides, and the phosphomannose isomerase (PMI) protein from *Escherichia coli*, which was used as a selectable marker during the transformation of DP51291 maize.

Prior to commercial approval, GM crops underwent a thorough environmental risk assessment (ERA) to determine potential for environmental harm resulting from cultivation. The ERA framework provides a rigorous, appropriate, and science-based tool for assessing potential risks associated with GM plants which include potential for weediness, gene flow, and risk to beneficial non-target organisms (NTOs).^18,19^ To facilitate the characterization of environmental risk, problem formulation can be used to develop hypotheses of potential harm by understanding the receiving environment, characteristics of the introduced trait, and biology of the crop.^18,20,21^ Potential for weediness and outcrossing is driven by the biology of the crop. Maize is an annual plant that only survives under favorable conditions and lacks seed dormancy limiting its survival over multiple growing seasons^22^ resulting in low potential for weediness. Maize also lacks sexually compatible relatives within maize cultivation environments that when combined with environmental (e.g., pollen viability,^23^ pollen dispersal,^24,25^ timing between flowering periods^26^) and genetic (i.e., ability to outcross and produce fertile progeny)^27^ barriers would not result in an increased risk for outcrossing. Therefore, based on what is known about the biology of maize, the receiving environment, and the intended traits of DP51291 maize, the primary focus of this ERA is on the potential effects on beneficial NTOs as there is low anticipated risk of gene flow or weediness for the IPD072Aa protein expressed in DP51291 maize.

Potential risk to beneficial NTOs that may interact with the plant (e.g., feeding on the plant or feeding on prey that have eaten the plant) within the agricultural ecosystem^28^ can be characterized as the ratio of an adverse effect (i.e., values) to an exposure estimate resulting in margins of exposure (MoE). For the DP51291 maize ERA, problem formulation was used to deduce pollinators and pollen feeders, soil-dwelling organisms, aquatic organisms, predators and parasitoids, insectivorous birds, and granivorous mammals may be functional groups of concern based on the receiving environment.

To assess hazard to the functional groups of concern, ERAs utilize a tiered testing strategy. Tier I studies are laboratory-based studies using laboratory reared species as surrogates that assess effects at the individual level, whereas higher tier studies (Tier II, Tier III, or Tier IV) are conducted either in a laboratory, greenhouse, or field to assess the potential for effects under increasingly realistic exposure conditions. Although higher tier studies are more realistic, confounding variables may occur and can complicate data interpretation.^28^

The IPD072Aa protein encoded by the *ipd072Aa* gene has previously been evaluated in the maize event DP-Ø23211–2 (hereafter referred to as DP23211),^8,29–31^ based on the framework described by the United States Environmental Protection Agency (US EPA)^32^ and by the USDA Animal and Plant Health Inspection Service (APHIS).^33^ DP23211 maize confers control of corn rootworms via two modes of action: the expression of a DvSSJ1 double-stranded RNA in addition to the IPD072Aa protein.^34^ When a new GM event in the United States is submitted by a registrant for commercial registration to undergo the US EPA and the USDA regulatory review processes, relevant new and existing information is submitted and reviewed to assess the potential risks associated with the new GM plant. In this case, the DP23211 event has previously been reviewed by the US EPA and deregulated (i.e., antecedent event) by the USDA, and the previously generated data, risk assessment, and regulatory decisions pertaining to the IPD072Aa protein expressed in DP23211 maize^8,29–31,35^ can be leveraged to facilitate streamlined risk assessments and regulatory reviews for the maize event, DP51291. This is accomplished through the use of problem formulation that can take into account the similarities and differences of the receiving environment, characteristics of the introduced trait, and biology of the crop to bridge the previous antecedent event risk assessment to the new event risk assessment. The USDA has previously utilized this approach to review requests and conduct a Plant Pest Risk Similarity assessment for the extension of an antecedent event’s nonregulated status to new event(s) that contain similar transgene(s) that encode the same protein(s) as the antecedent event.^36^

To determine if previously conducted hazard studies were protective (i.e., tested at high enough concentrations to address potential risk of DP51291 maize due to greater expression of the IPD072Aa protein compared to DP23211^8^) or if new studies were warranted, the results of the exposure assessment for DP51291 maize were analyzed in the context of previously conducted hazard studies to evaluate potential risk.^8^ The mode of action^17^ and specificity^37^ of the IPD072Aa protein were also considered to aid in directing the exposure and hazard assessment. Problem formulation identified data was needed to cover the surrogate species in the following functional groups: aquatic organisms (i.e., *Daphnia magna)*, soil-dwelling decomposers and detritivores (i.e., *Folsomia candida*), predators and parasitoids (i.e., *Chrysoperla rufilabris* and *Pediobius foveolatus*). This ERA is focused on the insecticidal IPD072Aa protein expressed in DP51291 maize and assesses the potential exposure and hazard to NTOs using both new and existing hazard studies,^8^ spectrum of activity characterization,^37^ and a supporting field assessment.

## Materials and Methods

### IDP072Aa Protein Expression in DP51291 Maize Tissues

Expression of the IPD072Aa protein was characterized for DP51291 maize and used to assess if sufficiently conservative IPD072Aa protein concentrations were used in Tier I laboratory toxicity tests previously conducted for DP23211 for the purpose of assessing safety of DP51291 maize. DP51291 maize was planted during the 2021 growing season at 6 sites in commercial maize-growing regions of the United States (one site in Iowa, Illinois, Indiana, Pennsylvania, and Texas) and Canada (one site in Ontario). Normal agronomic practices (e.g., irrigation, fertilization, herbicide, and pesticide applications) were applied uniformly to the entire trial area as needed. Different types of tissue samples (i.e., root, leaf, pollen, forage, and grain) were collected throughout the growing season from DP51291 maize plants. The IPD072Aa protein concentrations in various plant tissue samples were determined using quantitative enzyme-linked immunosorbent assay (ELISA) using methods described previously for the IPD072Aa protein.^8^ Protein concentrations determined by ELISA (Table 1) were then used to evaluate the potential NTO exposure to the IPD072Aa protein from the cultivation of DP51291 maize.

**Table 1.** Highest dry weight concentrations of the IPD072Aa protein in various tissue types from DP51291 maize.

Tissue Type	Growth Stage	ng IPD072Aa/mg Tissue Dry Weight		
		Maximum^a^	Mean^b^	Standard Deviation
Root	R1	330	180	85
Leaf	V9	140^†^	69^†^	33
Pollen	R1	7.1^†^	1.2	1.4
Forage	R4	88^†^	34^†^	20
Grain	R6	12^†^	4.1	3.6

^†^Mean and/or maximum values relevant for the exposure assessment of the various functional groups.

^a^The maximum concentrations of the IPD072Aa protein in relevant DP51291 maize tissues at any single field trial location and any growth stage were used for determining worst-case estimated environmental concentrations (EECs).

^b^The highest mean concentrations of the IPD072Aa protein in applicable DP51291 maize tissue across field trial locations and at any growth stage were used for determining refined EECs.

### Estimated Environmental Concentration (EEC) Calculations

The environmental exposure assessment considers the probability and degree to which NTOs will be exposed to the IPD072Aa protein from DP51291 maize. Worst-case EECs of the IPD072Aa protein in DP51291 maize were determined using worst-case assumptions (i.e., maximum concentrations of the IPD072Aa protein in relevant DP51291 maize tissues, Table 1) to determine potential exposure for NTOs, including pollinators and pollen feeders, soil-dwelling organisms, aquatic organisms, predators and parasitoids, granivorous mammals and insectivorous birds.

#### Pollinators and Pollen Feeders

In the agroecosystem, honey bees and non-target Lepidoptera can be found in habitats (e.g., agricultural fields) where flowering plants are abundant and thus be exposed to pollen from a variety of different plant species.^38^ Honey bee foraging distances to collect pollen and nectar normally range from a few hundred meters up to approximately 9.6 kilometers^39^ and have been reported to range up to about 13 kilometers when resources near the hive are scarce.^40^ Adult honey bees feed on pollen from a variety of different plant species as a major source of protein and consume nectar as a source of carbohydrates.^41,42^ As pollen is a primary food source, honey bees are likely to be present and actively foraging during maize pollen shed.^43^

Most non-target Lepidoptera larvae do not feed on pollen directly, but are indirectly exposed to pollen as they feed on host plants that have been dusted with maize pollen via wind dispersal within or near agricultural landscapes.^44^ In addition, the degree of potential exposure of non-target Lepidoptera to DP51291 maize pollen will depend on the presence of host plants in and adjacent to fields and maize pollen deposition rates. Therefore, the worst-case EEC for honey bees and non-target Lepidoptera was calculated based on the maximum pollen concentration of the IPD072Aa protein (Table 1).

#### Aquatic Organisms

Potential exposure of non-target aquatic organisms to insecticidal proteins in GM maize has been considered previously in the literature, and the movement of decomposing post-harvest tissues was identified as being the most likely route of exposure for aquatic organisms.^45^ The worst-case EEC for aquatic organisms to the IPD072Aa protein in DP51291 maize tissues was estimated using the US EPA standard agricultural field-farm pond model (also called the US EPA standard pond model^46^). The US EPA standard pond model provides estimates for predicting pesticide runoff concentrations and uses the assumptions that runoff from a 10-hectare (ha) field is deposited in a 1-ha pond (2 meters deep; equivalent to 20,000,000 L of water). This approach has previously been adapted to model a very conservative exposure scenario (i.e., protein being released from maize tissues in an agricultural water body) for aquatic NTOs to expressed proteins in GM crops.^47–49^ The worst-case EEC for aquatic organisms was calculated based on the highest mean whole plant tissue (i.e., forage R4 kernel dough stage, 24–28 days after silking, Table 1) concentration of the IPD072Aa protein.

#### Soil-Dwelling Decomposers and Detritivores

IPD072Aa protein in DP51291 maize may enter the soil environment through rhizodeposition before harvest or from post-harvest senescent maize tissues.^50,51^ Soil-dwelling decomposers and detritivores are most likely to be exposed to the IPD072Aa protein by consuming post-harvest senescent maize tissues that are incorporated into the soil.^52^ Thus, the worst-case EEC for soil-dwelling organisms that consume decomposing plant material was calculated based on the maximum concentration of the IPD072Aa protein in R4 forage tissue (Table 1), which represented an environmentally relevant yet conservative exposure route.

#### Predators and Parasitoids

A predator or parasitoid may be exposed to the IPD072Aa protein via consumption of prey that has previously consumed tissue from a DP51291 maize plant (tritrophic transfer). Because a predator does not feed directly on the maize plant, one factor to consider in the exposure assessment for predators or parasitoids is the amount of the IPD072Aa protein that transfers and accumulates in the prey. Secondary exposures via prey are influenced not only by the rates of ingestion, digestion and excretion of plant material by the prey,^53^ but also the stability of the IPD072Aa protein within the prey. Furthermore, the concentration of the IPD072Aa protein in the prey of predators or parasitoids will vary by prey species, prey developmental stage, and the concentration of protein in the plant parts the prey are feeding on. Previous research with other bacteria derived insecticidal proteins has demonstrated that herbivorous prey contains lower concentrations of insecticidal proteins than the plants they feed on. This has been documented in phloem-feeders (e.g., aphids),^54–57^ lepidopteran larvae,^55,56,58^ *Spodoptera exigua*, ^59^ and thrips^60^ with ranges of 0.025 to 0.35 times the insecticidal protein concentration depending on the species and life stage. However, one species of prey that has been documented to have high concentrations of insecticidal proteins is the spider mite (*Tetranychus urticae*) with ranges from 0.7 and 3.0 times the concentration of the insecticidal protein.^56–58^ Although spider mites may contain higher concentrations of insecticidal proteins, predators in maize fields are generalist feeders and do not rely on a single pest species for food.^53^ Thus, most predators would be highly unlikely to consume a diet consisting of only spider mites. In contrast, there are some coccinellid species in maize that have been documented to preferentially feed on spider mite eggs, such as *Stethorus punctillum*.^57,61^ Although they preferentially feed on spider mite eggs, exposure to concentrations of the IPD072Aa protein would likely be low in the spider mite eggs compared to the adult spider mites.^62^ Therefore, the worst-case EEC for predators and parasitoids was calculated based on the maximum concentration of the IPD072Aa protein in V9 leaf tissue (Table 1) and assumes: 1) that 100% of the protein in the GM plant transfers to the prey and then subsequently is transferred to the predator; and 2) the prey items are exposed to the maximum concentration of the IPD072Aa protein expressed in tissue (from any above-ground plant tissue and from any growth stage^63^).

To refine the EEC, it was assumed that 1) 20% of the protein^62^ in the GM plant transfers to the prey and then the predator; and 2) the prey are exposed to the greatest mean concentration of the IPD072Aa protein expressed in tissue (i.e., highest mean value from any above-ground plant tissue from any growth stage, Table 1).

#### Insectivorous Birds

Some wild birds are insectivorous and could be exposed to the IPD072Aa protein via prey that has previously consumed tissue from a DP51291 maize plant (tritrophic transfer). The factors that may limit the potential exposure of wild birds to the IPD072Aa protein via prey are discussed above for predators and parasitoids. For insectivorous birds, the worst-case EEC assumes: 1) that 100% of the IPD072Aa protein in the GM plant transfers to the prey and then subsequently to the wild bird; and 2) that prey items are exposed to the maximum concentration of the IPD072Aa protein expressed in tissue (from any above-ground plant tissue and from any growth stage). Therefore, the worst-case EEC for predators and parasitoids was calculated based on the maximum concentration of the IPD072Aa protein in V9 leaf tissue (Table 1).

#### Granivorous Mammals

Granivorous wildlife (e.g., rodents) may be exposed to the IPD072Aa protein by feeding on DP51291 maize grain. Several factors may limit the potential exposure of granivorous mammals to the IPD072Aa protein from DP51291 maize such as wild rodents which are unlikely to consume only maize grain, as they typically feed on a variety of cereal seeds. For granivorous mammals, a worst-case EEC assumes consumption of a diet that contains 73% maize grain,^62^ and is based on the maximum concentration of the IPD072Aa protein in maize grain (Table 1). A daily dietary dose (DDD) for wild rodents can be calculated,^48^ which accounts for food intake, body weight, and the IPD072Aa protein concentration in DP51291 maize grain.

### Hazard Assessment Bioassays

All hazard study bioassays were conducted in compliance with Good Laboratory Practice regulations as provided in US EPA 40 CFR part 160. For the IPD072Aa protein bioassays, the IPD072Aa protein was expressed in *Escherichia coli*, lyophilized, and characterized as described previously.^37^ The equivalence between the plant and *E. coli* -produced IPD072Aa protein was evaluated using methods previously described in Boeckman *et al* (2021).^8^ Sensitive surrogate species from the different functional groups were tested using a Tier 1 laboratory study design targeting maximum hazard concentrations that exceeded the relevant worst-case EECs by at least 10 whenever possible.

#### Surrogate Species Selection

Appropriate surrogate species were selected based on the exposure assessment, the specificity of the IPD072Aa protein,^37^ and practical considerations (e.g., availability of test species, amenability to testing, and availability of established, reproducible, and robust study methods).^64,65^ Based on the selection criteria above, the following 10 surrogate species were assessed: *Apis mellifera* (honey bee larvae and adults), *Daphnia magna*, *Folsomia candida* (springtail), *Chrysoperla rufilabris* (green lacewing), *Coleomegilla maculata* (pink spotted lady beetle), *Hippodamia convergens* (convergent ladybird beetle), *Dalotia coriaria* (rove beetle), *Pediobius foveolatus* (parasitic hymenoptera), *Colinus virginianus* (Northern bobwhite quail), and *Mus musculus* (mouse). These species represented the following functional groups: pollinators and pollen feeders, aquatic organisms, soil-dwelling decomposers and detritivores, predators and parasitoids, insectivorous birds, and granivorous mammals.

For the surrogate insect bioassays, an additional review was conducted to see if previously conducted hazard studies for the IPD072Aa protein were protective (i.e., tested at high enough concentrations to address potential risk of DP51291 maize due to greater expression of the IPD072Aa protein compared to DP23211 maize^8^), or if new studies were warranted. This review consisted of utilizing the DP51291 maize IPD072Aa protein expression data to determine the updated EECs. Based on the DP23211 hazard study endpoints^8^ and DP51291 maize-specific EECs, DP51291 maize MoEs were then estimated. For instances where the MoEs were less than 10 for a specific surrogate species, new hazard studies were conducted for DP51291 maize. MoE calculation methods are described in more detail in the Margin of Exposure Calculations section.

The review identified data needs to cover the surrogate species in the following functional groups: aquatic organisms (i.e., *Daphnia magna)*, soil-dwelling decomposers and detritivores (i.e., *Folsomia candida*), predators and parasitoids (i.e., *Chrysoperla rufilabris* and *Pediobius foveolatus*). Other functional group hazard studies are described elsewhere by Boeckman *et al*. (2019)^37^ and Boeckman *et al*. (2021),^8^ and a summary of the bioassays and associated endpoints can be found in Table 2. For each bioassay conducted, the surrogate species were exposed to concentrations that targeted the maximum hazard dose of at least 10 times the worst-case EECs whenever possible. Further, the no-observed-effect concentration (NOEC), no-observed-effect dose (NOED), or no-observed-effect dietary dose (NOEDD) were determined for the appropriate endpoints (i.e., mortality, adult weight) for the IPD072Aa protein (Table 2).

**Table 2.** Tier I laboratory studies characterizing effects of the IPD072Aa protein on representative surrogate non-target organisms.

Functional Group	Species (Common Name)	Class Order Family	Guideline	Study Design	Concentrations	Endpoints Assessed	Hazard Study Observations	Hazard Study Endpoint
*Pollinators and Pollen Feeders*	*Apis mellifera* (honey bee larvae)^a^	Insecta Hymenoptera Apidae	OECD GD No. 239	22-day: IPD072Aa protein incorporated in diet	Target of 100 and 200 ng IPD072Aa protein/larva	Larval & pupal survival, adult emergence, weight at adult emergence	No effects on larval survival, pupal survival, adult emergence, or adult weight at emergence.	**NOED =** **200 ng** **IPD072Aa protein/larva**
	*Apis mellifera* (honey bee adult)^a^		OECD 245	10-day: IPD072Aa protein incorporated in diet	Mean daily dose of 640 and 1300 ng IPD072Aa protein/bee/day	Survival, adult body weight	No effects on adult body weight or survival.	**NOEDD =** **1300 ng IPD072Aa protein/bee/day**
	Non-target Lepidoptera	Tier I hazard studies were not conducted based on negligible potential for exposure and lack of effects demonstrated in the spectrum of activity characterization.^37^						
*Aquatic Organisms*	*Daphnia magna* (water flea)	Branchiopoda Diplostraca Daphniidae	OECD 211	21-day static renewal: IPD072Aa protein incorporated into water	Target of 15 mg IPD072Aa protein/L	Survival, reproduction	No adverse effects on survival or reproduction.	**NOEC =** **15 mg** **IPD072Aa/L**
*Soil Dwelling Decomposers & Detritivores*	*Folsomia candida* (springtail)	Collembola Collembola Isotomidae	–	28-day: IPD072Aa protein incorporated into diet	Target of 4000 ng IPD072Aa protein/mg diet	Survival, reproduction	No adverse effect on reproduction and no effects on survival.	NOEC = 4000 ng IPD072Aa protein/mg diet
*Predators & Parasitoids*	*Chrysoperla rufilabris* (green lacewing)	Insecta Neuroptera Chrysopidae	–	21-day: IPD072Aa protein incorporated in diet	Target of 2000 ng IPD072Aa protein/mg diet	Survival, pupation	No biologically relevant adverse effects on survival or pupation.	**NOEC =** **2000 ng IPD072Aa protein/mg diet**
*Predators & Parasitoids (cont.)*	*Coleomegilla maculata* (pink spotted lady beetle) ^a^	Insecta Coleoptera Coccinellidae	–	28-day: IPD072Aa protein incorporated in diet	Target of 100, 500, and 1000 ng IPD072Aa protein/mg diet	Survival, weight, days to adult emergence	No adverse effects on survival, weight, or number of days to adult emergence at 100 ng IPD072Aa protein/mg diet.	**NOEC =** **100 ng IPD072Aa protein/mg diet**
	*Hippodamia convergens* (convergent ladybird beetle) ^a^	Insecta Coleoptera Coccinellidae	–	28-day: IPD072Aa protein incorporated in diet	Target of 100, 500, and 1000 ng IPD072Aa protein/mg diet	Survival, weight, days to adult emergence	No adverse effects on survival at 500 ng IPD072Aa protein/mg.	**NOEC =** **500 ng IPD072Aa protein/mg diet**
	*Pediobius foveolatus* (parasitic hymenoptera)	Insecta Hymenoptera Eulophidae	–	7-day: IPD072Aa protein incorporated into diet	Target 2000 µg IPD072Aa protein/ml diet	Survival	No adverse effects on survival.	**NOEC =** **2000 µg** **IPD072Aa protein/ml diet**
***Insectivorous Birds***	*Colinus virginianus* (northern bobwhite quail) ^a^	Aves Galliformes Odontophoridae	OCSPP Guideline 850.2100	14-day limit dose	Limit dose of 2,000 mg IPD072Aa protein/kg body weight	Survival, body weight, food consumption, behavior	No mortality, abnormal behavior or signs of toxicity.	**NOEC = 2000 mg IPD072Aa protein/kg body weight** **LD**_**50**_ ** > 2000 mg IPD072Aa protein/kg body weight**
***Granivorous Mammals***	*Mus musculus* (mouse) ^a^	Mammalia Rodentia Muridae	OECD GD for Testing of Chemicals, Section 4 (Part 423)	14-day: Acute oral assay with IPD072Aa protein	Limit dose of 2,000 mg IPD072Aa protein/kg body weight	Survival, acute oral toxicity (based on evaluation of body weight, clinical signs, and gross pathology)	No mortality or other evidence of acute oral toxicity was observed, based on evaluation of body weight, clinical signs, and gross pathology.	**LD**_**50**_ ** > 2000 mg IPD072Aa protein/kg body weight**

Note: median lethal concentration (LD_50_), no-observed-effect concentration (NOEC), no-observed-effect dose (NOED), or no-observed-effect-dietary dose (NOEDD).

^a^Study was conducted previously for IPD072Aa,^8^ and the associated hazard studies and endpoints are summarized accordingly.

Depending on the organism of interest, bioassays were conducted in environmental chambers, with temperatures ranging from 20°C to 25°C, light regimes maintained either at continuously dark or with a 16-hour light:8-hour dark photoperiod, and relative humidity maintained above 65% for the insect bioassays. Bioassay tests are briefly described below for the functional group set of studies identified to cover the additional data needs.

#### Aquatic Organisms

*D. magna* juveniles ( < 24-hours) were exposed to an untreated control and a IPD072Aa protein concentration of 15 mg IPD072Aa protein/L under static-renewal conditions in glass beakers for 21-days. There were 10 replicates for both the control and treatment with the IPD072Aa protein. Test conditions and endpoint analysis of survival and reproduction (Supplemental Table 1, Table 2) were based on OECD guidance.^66^

#### Soil-Dwelling Decomposers and Detritivores

Springtail (*F. candida*) adults were exposed to an untreated diet, a positive control diet containing 1000 ng teflubenzuron/mg diet dry weight, or a diet containing 4000 ng IPD072Aa protein/mg diet dry weight for 28-days. Each treatment consisted of 8 replicates of 10 individuals housed in small wide-mouth glass jars. After 28 days, the endpoints assessed were adult mortality and reproduction (Supplemental Table 1, Table 2).

#### Predators and Parasitoids

Green lacewing (*C. rufilabris*) neonates were exposed to an untreated diet, a positive control diet (25000 ng cryolite/mg diet wet weight), or a diet containing 2000 ng IPD072Aa/mg diet dry weight every other day for 21 days in 30 mL plastic cups. Each treatment had 40 replicates. The endpoints assessed included survival and pupation (Supplemental Table 1, Table 2).

Parasitoid wasp (*P. foveolatus*) adults were exposed to an untreated control, a positive control (20,000 µg boric acid/ml sucrose diet), or 2000 µg IPD072Aa protein/ml diet in 30% sucrose. *P. foveolatus* were housed in 30 mL plastic cups (30 replicates per treatment) and were provided diet containing one of the treatments mentioned above every other day for 7 days. On completion of the 7-day bioassay, the endpoint assessed was survival (Supplemental Table 1, Table 2).

### Field Assessment of Non-Target Arthropods

The field portion of the study was conducted during the 2022 growing season at three field sites in commercial maize-growing regions of the United States (one site in Illinois, Iowa, and Pennsylvania).

A randomized complete block design with three blocks was utilized at each site. Each block included DP51291 maize and control maize planted in 40-row plots at a rate of 150 seeds per row, except for the Germansville, PA site, where DP51291 maize was planted in 37–38 row plots and control maize was planted in 29–38 row plots. Each row was 30 m (98.4 ft) in length with 76 cm (30 in.) between rows. Each block was separated by an alley of approximately 1 m (36 in.) in width. The entire field trial was surrounded by at least 4 external border rows. As needed and at a given site, maintenance products were uniformly applied to all plots to minimize weed and disease pressure.

Three sampling methods (visual, sticky traps, and pitfall traps) were used to monitor nontarget arthropods (NTAs). To minimize edge effects, sampling was conducted near the center of each plot and remained fixed for the duration of the study. Within the sampling area of each plot, data were collected from three randomly assigned sampling points, which remained fixed for all four evaluation stages.

Data were collected at the V4-V6, V8-V10, 50% pollen shed, and R2-R3 growth stages from each plot of DP51291 maize and control maize. Visual observation counts were identified and recorded at the field locations. Specimens collected in the sticky traps and pitfall traps were returned to the laboratory for identification and counts.

### Statistical Analyses

#### Hazard Bioassays

Statistical analyses for *F*. *candida*, *C. rufilabris* and *P. foveolatus* bioassays were conducted using SAS™ software, Version 9.4^67^ separately for each study and each measured endpoint. Statistical analysis of survival data was conducted using Fisher’s exact test (SAS PROC MULTTEST) to determine if the survival observed for the IPD072Aa protein treatments were less than the survival observed with the untreated control treatment included in each study. The statistical analysis methods used to assess the weight of surviving insects were dependent upon the validity of statistical assumptions for each data set. For some experiments, the normality assumption was satisfied by the data distributions of the test and control entries, thus an analysis of variance (SAS PROC GLIMMIX) was conducted to assess if the test diet caused growth inhibition. For reproduction, a generalized linear mixed model (SAS PROC GLIMMIX) was fit to the reproduction data assuming a Poisson distribution and a fixed effect of treatment. For each of these tests, all *P*-values were considered significant if *p* < 0.05.

Statistical analyses for the *D. magna* bioassay were conducted using CETIS Version 1.9.^68^ At the termination of the study, data obtained on organism survival and reproduction were statistically analyzed to identify significant treatment-related effects. Survival was analyzed with a Fisher’s Exact Test. In the case where a significant dose-response could not be determined, the NOEC was empirically derived. All statistical analyses were conducted at the 95% level of certainty.

Statistical analyses for the remaining species were conducted for each study and each measured endpoint as previously described.^8^

#### Field Assessment of Non-Target Arthropods

Statistical analyses were conducted to evaluate and compare abundance data and diversity indices of DP51291 maize to the control maize. Taxa data were collected using three sampling methods (visual observations, sticky traps, and pitfall traps) from three sites at four evaluation times (V4-V6, V8-V10, 50% pollen shed, and R2R3 growth stages). Statistical analyses were conducted separately for each sampling method at each site using SAS software, Version 9.4 (SAS Institute Inc.).

For abundance data, arithmetic average of data from multiple sampling points in each plot was calculated and used as the plot-level abundance for statistical analysis. For a given taxon and sampling method at a given evaluation time, if both entries had two or more plots with non-zero abundance; and the average plot-level abundance across blocks for the negative control entry was 3 or greater with sticky traps or pitfall traps, or 0.3 or greater with visual observations, then a linear mixed model analysis was conducted; otherwise, no statistical analysis was conducted.

Linear mixed model analyses on abundance data utilized the following model:

$$\log(y_{\mathrm{bdt}}+1)=\mu +B_{b}+T_{d}+S_{t}+ST_{\mathrm{dt}}+\varepsilon_{\mathrm{bdt}}$$

$y_{\mathrm{bdt}}$ represents the abundance at the *t*^th^ evaluation time in the plot designated to the *d*^th^ entry in the *b*^th^ block. The natural logarithm of the plot-level abundance plus one was modeled as the response. Parameter μ denotes the overall mean, $T_{d}$ denotes the effect of the *d*^th^ entry, $S_{t}$ denotes the effect of the *t*^th^ evaluation time, and $ST_{\mathrm{dt}}$ denotes the effect of interaction between the *d*^th^ entry and the *t*^th^ evaluation time (entry-by-time interaction). All the above parameters were modeled as fixed effects. Parameter $B_{b}$ denotes the effect of the *b*^th^ block, and $\varepsilon_{\mathrm{bdt}}$ denotes the error term. It was assumed that the random block effect $B_{b}$ is independently identically distributed (i.i.d.) according to the normal distribution with mean zero and variance $\sigma_{B}^{2}$, *i.e*., $B_{b}∼\mathrm{iidN}\left(0,\sigma_{B}^{2}\right)$; the random errors $\varepsilon_{\mathrm{bdt}}$ is denotes the error term with $\varepsilon_{\mathrm{bdt}}∼\mathrm{iidN}\left(0,\sigma_{e}^{2}\right)$.

When only one evaluation time met the minimum abundance criterion, analysis was conducted using the following model:

$$\log(y_{\mathrm{bd}}+1)=\mu +B_{b}+T_{d}+\varepsilon_{\mathrm{bd}}$$

$y_{\mathrm{bd}}$ represents the abundance in the plot designated to the *d*^th^ entry in the *b*^th^ block. Parameter μ denotes the overall mean, $T_{d}$ denotes the effect of the *d*^th^ entry (fixed effect), $B_{b}$ denotes the effect of the *b*^th^ block (random effect, $B_{b}∼\mathrm{iidN}\left(0,\sigma_{B}^{2}\right)$), and $\varepsilon_{\mathrm{bd}}$ denotes the error term with $\varepsilon_{\mathrm{bd}}∼\mathrm{iidN}\left(0,\sigma_{e}^{2}\right)$

To evaluate taxa diversity, three diversity indices were calculated separately for each sampling method: Shannon’s diversity index, Pielou’s evenness index, and Simpson’s diversity index. When there is only one species, Shannon’s index exactly equals zero. Furthermore, high values of the Shannon’s index would be representative of a more diverse environment. Pielou’s evenness index normalizes the Shannon index to a value between 0 and 1, and lower values indicate less evenness of taxa proportions and higher values indicate more evenness. The Simpson’s diversity index has a value between 0 and 1, and higher values indicate more diversity while lower values indicate less diversity.

For taxa that met the minimum abundance criteria, an across stage analysis was conducted. Linear mixed model analyses on the diversity indices were conducted with the assumption that the random block effect is independently identically distributed according to the normal distribution. SAS PROC MIXED with the default restricted maximum likelihood (REML) method was used to estimate the variance components and provide statistical inference. The approximated degrees of freedom for the statistical test were derived using the Kenward-Roger method.^69^ The estimated means are known as empirical best linear unbiased estimators (hereafter referred to as LSMeans). A significant difference was identified if a P-value was < 0.05.

The statistical results for transformed data were backtransformed to the original data scale for reporting purposes. The following statistical results were reported: LS-Means, 95% confidence intervals and *P*-values.

### Margin of Exposure Calculations

For each surrogate species laboratory study, the NOED or NOEC was determined for the respective endpoints (i.e., mortality or weight gain) for the IPD072Aa protein and compared to the worst-case EEC (or refined EEC where applicable) to determine the MoE. For example, the NOED hazard value was divided by the EEC to obtain the corresponding MoE. Tier I testing typically targets a maximum hazard dose greater than 10 times the EEC as the resulting MoEs are considered highly conservative and indicate minimal risk under relevant environmental conditions.^53^ MoE values less than 10 are not necessarily indicative of risk, but rather reflect potential uncertainties in the risk assessment that may need to be accounted for through additional exposure refinement or testing.^53^ Current guidance indicates more than 50% mortality during Tier I screening is required to trigger higher tier testing.^53,70^ The MoEs in this ERA are based on NOECs or NOEDs rather than a 50% mortality effect which adds an additional layer of conservatism in the risk assessment. However, if effects are observed during the hazard surrogate laboratory testing, the magnitude of effects could be placed in the context of current guidance^53,70^ to determine whether additional testing is needed. Given the continuous dietary exposure conducted with Tier I screening studies, a level of concern of 1 (i.e., NOEC/EEC = 1) is typically used to identify potential risk.^53^

For the current assessment, cases where the MoEs (i.e., NOEC/EEC or NOED/EEC) were less than 10, potential uncertainty was addressed through the refinement of EECs through inclusion of more realistic environmental conditions and ecological processes that more closely reflect actual NTO exposure in the field. All MoE calculations were conducted based on the dry weight concentration of the IPD072Aa protein in DP51291 maize tissues as the tissue samples were lyophilized prior to protein expression analyses. The dry weight concentrations are considered high estimates and add a layer of conservatism to the assessment, since in reality NTOs would be exposed to levels comparable to fresh weight levels.^71^

## Results and Discussion

The exposure assessment considers the degree to which NTOs will be exposed to the IPD072Aa protein from DP51291 maize. Conservative EECs of the IPD072Aa protein in DP51291 maize were determined using worst-case assumptions (i.e., maximum concentrations of the IPD072Aa protein in relevant DP51291 maize tissues) to determine potential exposure for NTOs, including pollinators and pollen feeders, soil-dwelling organisms, aquatic organisms, predators and parasitoids, granivorous mammals and insectivorous birds. These worst-case EECs were used to calculate estimated MoEs using hazard data from Tier I laboratory bioassays described below. In cases where MoEs for NTOs based on worst-case EECs were less than the threshold of 10, refined EECs were calculated to reflect more realistic environmental conditions and ecological processes that reduce actual NTO exposure under field conditions.

### Pollinators and Pollen Feeders

One key consideration in the exposure assessment of pollen feeders and pollinators is the concentration of the IPD072Aa protein in DP51291 maize pollen. These organisms can be exposed to the IPD072Aa protein by directly feeding on DP51291 maize pollen or by incidentally feeding on pollen that may have been deposited on host plant leaves. In a comprehensive field expression study, the mean concentration was 1.2 ng IPD072Aa protein/mg pollen with a maximum concentration of 7.1 ng IPD072Aa protein/mg pollen (Table 1). For non-target pollinators and pollen feeders, the worst-case EEC assumes that pollen feeders and pollinators are exposed to the maximum concentration of IPD072Aa protein in pollen.

#### Honey Bees

Hazard studies were previously performed^8^ to assess the dietary effect of the IPD072Aa protein on honey bee larvae and adults. For honey bee larvae, no effects on larval survival, pupal survival, adult emergence, or adult weight at emergence were observed up to concentrations of 200 ng IPD072Aa protein/larva, thus the NOED was 200 ng IPD072Aa protein/larva. For adult honey bees, no effects on adult body weight or survival were observed up to 1300 ng IPD072Aa protein/bee/day, thus the NOEDD was 1300 ng IPD072Aa protein/bee/day (Table 2).

The amount of pollen consumed by one honey bee larva has previously been estimated to be approximately 1.5 - 2.0 mg over the course of development,^72^ and the amount of pollen consumed by one honey bee adult has previously been estimated to be 3.4 - 4.3 mg per day.^43^ Based on the maximum concentration of the IPD072Aa protein in DP51291 maize pollen (i.e., 7.1 ng IPD072Aa protein/mg, Table 1), the worst-case EEC for honey bee larvae and chronic adult exposed to the IPD072Aa protein in DP51291 maize pollen is 14.2 ng/per larvae dry weight and 30.5 ng per bee per day dry weight, respectively (Table 3). A refined EEC was not determined for honey bees because the MoE using the worst-case assumptions indicated low risk. Many factors will further reduce actual exposure below the worst-case EECs, including the degree of spatial overlap of honey bee foraging ranges with DP51291 maize fields as well as the degree of temporal overlap of maize anthesis with sensitive insect life stages. Furthermore, the dry weight concentrations are considered high estimates as they are not influenced by the water content of plant tissues, and the NTOs would be exposed to comparable fresh weight levels.^71^ Lastly, population level exposure to DP51291 maize pollen is expected to be low as maize pollen is not expected to be the only type of pollen or dietary component consumed by honey bees.

**Table 3.** Worst-case and refined estimated environmental concentrations (EEC) and margin of exposure (MoE) for representative non-target organisms (NTOs) exposed to IPD072Aa protein from DP51291 maize.

Functional Group	Species (Common Name)	Worst-case EEC	Refined EEC	Hazard Study Endpoint	MoE for DP51291 Maize ^c^
*Pollinators and Pollen Feeders*	*Apis mellifera* (honey bee larvae)	Larvae eat 2 mg of pollen: Maximum pollen concentration EEC = 14.2 ng/mg	No refinement to worst-case EEC considered	NOED = 200 ng IPD072Aa protein/larva^a^	14
	*Apis mellifera* (honey bee adult)	Assume adults consume 4.3 mg of pollen: Maximum pollen concentration EEC = 30.5 ng/mg	No refinement to worst-case EEC considered	NOEDD = 1300 ng IPD072Aa protein/bee/day^a^	42.6
	Non-target Lepidoptera	Maximum pollen concentration EEC = 7.1 ng/mg	No refinement to worst-case EEC considered	NOEC = 1000 ng/IPD072Aa protein/mg diet^b^	140.8
*Aquatic Organisms*	*Daphnia magna* (water flea)	EEC = 0.383 mg/L	No refinement to worst-case EEC considered	NOEC = 15 mg IPD072Aa/L	39
*Soil Dwelling Decomposers & Detritivores*	*Folsomia candida* (springtail)	EEC = 88 ng/mg	No refinement to worst-case EEC considered	NOEC = 4000 ng IPD072Aa protein/mg diet	45.5
*Predators & Parasitoids*	*Chrysoperla rufilabris* (green lacewing)	100% trophic transfer of the maximum concentration in above ground tissue (any growth stage) EEC = 140 ng/mg		NOEC = 2000 ng IPD072Aa protein/mg diet	14.3
	*Coleomegilla maculata* (pink spotted lady beetle)		20% trophic transfer of the mean concentration in aboveground tissue (highest mean concentration in above ground tissue across the growing season). EEC = 13.8 ng/mg	NOEC = 100 ng IPD072Aa protein/mg diet ^a^	0.71 7.3 - refined
	*Hippodamia convergens* (Convergent ladybird beetle)			NOEC = 500 ng IPD072Aa protein/mg diet ^a^	3.57 36 - refined
	*Pediobius foveolatus* (parasitic hymenoptera)			NOEC = 2000 µg IPD072Aa protein/ml diet	14.3
***Insectivorous Birds***	*Colinus virginianus* (northern bobwhite quail)	100% trophic transfer of the maximum concentration in above ground tissue (any growth stage) EEC = 140 ng/mg	No refinement to worst-case EEC considered	NOEC = 2000 mg IPD072Aa protein/kg body weight ^a^ LD_50_: > 2000 mg IPD072Aa protein/kg body weight ^a^	14.3
***Granivorous Mammals***	*Mus musculus* (mouse)	The worst-case EEC for wild mammals is based on the daily dietary dose (DDD) via consumption of R6 grain. EEC = 2.891 mg/kg body weight	No refinement to worst-case EEC considered	LD_50_: > 2000 mg IPD072Aa protein/kg body weight ^a^	691.8

Note: median lethal concentration (LD_50_), no observed effect concentration (NOEC), no observed effect dose (NOED), or no observed effect dietary dose (NOEDD).

All MoEs are calculated based on tissue dry weight (DW). The dry weight concentrations are considered high estimates, since in reality NTOs would be exposed to levels comparable to fresh weight levels^71^^a^Hazard study endpoint from Tier I studies previously conducted for IPD072Aa.^8^^b^ Hazard study endpoint from the spectrum of activity characterization that was previously conducted for the IPD072Aa protein.^37^^c^Based on worst-case EEC unless otherwise stated that it is based on a refined EEC.

The larval honey bee NOED was 200 ng IPD072Aa protein/larvae (Table 2), and the corresponding MoE, for honey bee larvae exposed to the IPD072Aa protein in DP51291 maize pollen is 14 (Table 3). The adult honey bee NOEDD was 1300 ng IPD072Aa protein/bee (Table 2), and the MoE for honey bee adults exposed to the IPD072Aa protein in DP51291 maize pollen is 42.6 (Table 3). Based on the Tier I hazard assessments and the MoE values, there is support for the conclusion that risk to honey bees is negligible. This conclusion for honey bee larvae and adults is consistent with the previous assessment for the IPD072Aa protein in the antecedent maize event, DP23211.^8^ Therefore, no unreasonable harm on honey bee larvae or adults is anticipated from the IPD072Aa protein in DP51291 maize at environmental relevant concentrations.

#### Non-Target Lepidoptera

Based on the concentration of the IPD072Aa protein in D51291 maize pollen, the worst-case exposure of non-target Lepidoptera to the IPD072Aa protein was estimated to be 7.1 ng IPD072Aa protein/mg pollen (Table 3). This worse-case exposure EEC analysis assumes that non-target Lepidoptera are only consuming pollen and does not consider the many factors that can reduce the actual exposure to the IPD072Aa protein from DP51291 maize pollen (e.g., field margin weed management practices to decrease host plant density,^73^ maize pollen deposition rates^25,74,75^ and host plant characteristics, environmental conditions (i.e., heat, relative humidity,^76^ and ultra-violet radiation^77^ may weaken the integrity of the pollen capsule and protein stability, temporal and spatial overlap to DP51291 maize, and/or feeding behavior). As part of the IPD072Aa protein spectrum of activity characterization, four Lepidoptera species [i.e., European corn borer (*Ostrinia nubilalis* Hubner Lepidoptera: Crambidae, codling moth (*Cydia pomonella* Linnaeus, Lepidoptera: Tortricidae), painted lady (*Vanessa cardui* Linnaeus, Lepidoptera Nymphalidae), and corn earworm (*Helicoverpa zea* Boddie, Lepidoptera: Noctuidae)], were assessed to test for cross-order activity. No adverse effects on survival were observed at 1000 ng IPD072Aa protein/mg diet^37^ for any of the tested species, which is a concentration that is approximately 140.8X the worst-case EEC for non-target Lepidoptera (Table 3). Considering the lack of activity of the IPD072Aa protein observed on Lepidoptera as well as negligible potential for exposure, the IPD072Aa protein in DP51291 maize is unlikely to be harmful to non-target Lepidoptera at environmentally realistic concentrations. This conclusion for non-target Lepidoptera is consistent with the previous assessment for the IPD072Aa protein in the antecedent maize event, DP23211.^8^ Therefore, potential risk is negligible and no further exposure refinements were necessary.

### Aquatic Organisms

Aquatic habitats may be near agricultural areas; however, exposure of aquatic organisms to GM crops is limited temporally and spatially^78^ with the movement of decomposing tissue being identified as the most likely route of exposure.^45^ Furthermore, exposure to aquatic organisms to GM maize has been demonstrated to be very small.^79^ To help inform the risk assessment for aquatic organisms, the specificity and environmental fate of the protein and worst-case assumptions about the potential entry of maize tissue into the aquatic environment can be used.^47^

The worst-case EEC for aquatic organisms was calculated based on the greatest mean whole plant tissue concentration of the IPD072Aa protein (i.e., 34 ng IPD072Aa protein/mg in forage R4 tissue; Table 1). Based on the assumptions of the US EPA farm pond model, the worst-case EEC for aquatic organisms is 0.383 mg/l for the IPD072Aa protein (Table 3). Although these worst-case assumptions are extremely conservative, the US EPA has previously noted that more refined exposure estimates are generally not needed unless this screening level calculation indicates exposure levels above a level of concern.^71^ The worst-case EEC is conservative in that not all the maize plant biomass would be transported into an adjacent aquatic system. In addition, not all the IPD072Aa protein would be freely soluble and immediately available to aquatic organisms which also have other food choices besides maize forage tissue. The IPD072Aa protein would also be expected to degrade by abiotic factors (i.e., photodegradation, pH, and temperature) and by biotic factors (i.e., microbial degradation) due to its protein nature, thus actual exposure would likely be significantly less.^80,81^ Further, field surveys have demonstrated that insecticidal proteins in headwater streams may be present in low concentrations (i.e., ng/L) which is much less than the worst-case EEC for the IPD072Aa protein (i.e., mg/L).^82^ Therefore, a refined EEC was not determined for aquatic organisms because the worst-case EEC indicates low potential risk.

In the *D. magna* hazard study, no adverse effect on daphnia reproduction or survival were observed at the concentration tested (i.e., 15 mg IPD072Aa protein/L, Supplemental Table 1, Table 2). Therefore, the NOEC was determined to be 15 mg IPD072Aa protein/L, and the corresponding MoE is 39 (Table 3).

Based on previously discussed conservative assumptions used to estimate aquatic exposure, additional mitigation factors (i.e., protein degradation), and overall MoE value, potential risk to non-target aquatic organisms is negligible. This conclusion for aquatic organisms is consistent with the previous assessment for the IPD072Aa protein in the antecedent maize event, DP23211.^8^ Therefore, no unreasonable harm is anticipated to aquatic organisms from the IPD072Aa protein in DP51291 maize at environmentally realistic concentrations.

### Soil-Dwelling Decomposers and Detritivores

Soil-dwelling detritivores and decomposers will most likely consume maize tissue that is incorporated into the soil post-harvest.^78^ Springtails were selected to represent a non-target detritivore and were assessed to evaluate the dietary effect of the IPD072Aa protein on survival and reproduction. No adverse effects were observed at the limit concentration tested of 4000 ng IPD072Aa protein/mg diet (Supplemental Table 1, Table 2). Therefore, based on a NOEC of 4000 ng IPD072Aa protein/mg diet, the MoE is 45.5 (Table 3). As soil-dwelling detritivores can also be exposed to the IPD072Aa protein through rhizodeposition in the root zone, the root tissue with the highest protein expression was also assessed for conservatism in this ERA. Using the NOEC of 4000 ng IPD072Aa protein/mg diet and the maximum concentration of the IPD072Aa protein in root tissue (Table 1), the MoE is 12.1 (Table 3). Further, as most soil detritivores and decomposers are typically located within the topsoil and insecticidal proteins degrade quickly,^50,83,84^ the forage tissue represents the most environmentally realistic exposure to soil-dwelling decomposers and detritivores. Based on the Tier I hazard test and the overall MoE value using forage tissue, a determination of no unreasonable harm on non-target soil dwelling organisms is anticipated from the IPD072Aa protein in DP51291 maize at environmentally relevant concentrations. These findings are further supported with the previous conclusion for the IPD072Aa protein in the antecedent maize event, DP23211,^8^ and with other works that have found no impact on soil invertebrate taxa from insecticidal proteins.^83,85,86^

### Predators and Parasitoids

A predator or parasitoid may be exposed to the IPD072Aa protein via consumption of prey that has previously consumed tissue from a DP51291 maize plant (tritrophic transfer). Because a predator does not feed directly on the maize plant, one factor to consider in the exposure assessment for predators or parasitoids is the amount of the IPD072Aa protein that transfers and accumulates in the prey. Secondary exposures via prey are influenced not only by the rates of ingestion, digestion and excretion of plant material by the prey,^53^ but also by the stability of the IPD072Aa protein within the prey.

The worst-case EEC for predators and parasitoids assumes: 1) that 100% of the protein in the GM plant transfers to the prey and then subsequently is transferred to the predator; and 2) the prey items are exposed to the maximum concentration of the IPD072Aa protein expressed in tissue (maximum from any above-ground plant tissue and from any growth stage). Based on these assumptions, the worst-case EEC is 140 ng/mg for the IPD072Aa protein (Table 3). The refined EEC assumes: 1) that 20% of the protein^62^ in the GM plant transfers to the prey and is transferred to the predator; and 2) that prey items are exposed to the greatest mean concentration of the IPD072Aa protein expressed in tissue (highest mean value from any above-ground plant tissue and from any growth stage). Based on these assumptions, the refined EEC is 13.8 ng/mg for the IPD072Aa protein (Table 3). These assumptions provide a realistically conservative refined EEC as predators in maize fields are generalist feeders and do not rely on a single pest species for food,^53^ many prey will not have been exposed to the greatest mean concentration of protein, many lepidopteran larvae will likely contain less than 20% of the protein,^55,56,62^ and lepidopteran eggs and aphids would have substantially reduced amounts of protein compared to the lepidopteran larvae. Several additional factors (e.g., protein degradation within prey species, not all prey items will have fed on GM plant, and/or protein dry weight concentrations are considered high estimates compared to actual fresh weight values, etc.) will reduce the actual exposure of predators and parasitoids to the IPD072Aa protein below the worst-case EEC, therefore the refined EEC represents a more environmentally realistic, but still conservative, exposure for predators and parasitoids.

#### Green Lacewing

*C. rufilabris* larvae were exposed to diet containing a target concentration of 2000 ng IPD072Aa protein, and no adverse effects on survival or pupation rate of green lacewing were observed (Table 2). There was a significant increase observed in days to pupation in the treatment group compared to the control (Supplemental Table 1). The delay in pupation observed in green lacewing larvae represented less than a 50% effect relative to the control. Therefore, the results indicate minimal risk and are not considered biologically meaningful due to typical trigger values for advancing to a Tier II study commonly set to a 50% effect level.^53^ Therefore, the NOEC was determined to be 2000 ng IPD072Aa protein/mg diet and the corresponding MoE for predators exposed to the IPD072Aa protein in DP51291 maize via prey is 14.3 (Table 3).

#### Pink Spotted Lady Beetle

*C. maculata* previously demonstrated adverse effects on survival, weight, and number of days to adult emergence at 500 and 1000 ng IPD072Aa protein/mg diet (survival, weight, and days to emerge were reduced by less than 50% at these higher concentrations).^37^ No adverse effects on survival, weight, or number of days to adult emergence of *C. maculata* were observed at 100 ng IPD072Aa protein/mg diet (Table 2). Therefore, the NOEC was reported as 100 ng IPD072Aa protein/mg diet,^8^ and the corresponding MoE for predators and parasitoids exposed to the IPD072Aa protein/mg in DP51291 maize via prey is 0.71 using worst-case exposure assumptions and 7.3 using refined exposure values (Table 3). Although the MoE is less than 10X, the *C. maculata* study represents a chronic exposure scenario. For chronic exposures, guidance indicates that NOECs less than 1X can indicate potential environmental risk.^53^ Based on the results of the hazard study and the refined EEC and MoE, no potential risk is anticipated as the NOEC is 7.3X the refined EEC. Furthermore, the significant differences observed in the *C. maculata* hazard study at 500 and 1000 ng IPD072Aa protein/mg diet are not likely to represent potential adverse effects at the population level as no significant hazard (i.e., less than 50% mortality^53^ was observed at 10-fold higher laboratory exposures. Therefore, the potential risk of adverse effects at environmentally relevant concentrations is unlikely.

Several factors will reduce the actual exposure of coccinellid species to the IPD072Aa protein via the predator route below the worst-case EECs. These factors include: 1) food and prey choice; 2) degradation of the IPD072Aa protein in prey; and 3) fresh weight versus dry weight concentrations.

Food and prey choice is a large component of the discussion regarding exposure reduction to coccinellid species to the IPD072Aa protein. As noted previously, previous research with other bacteria derived insecticidal proteins has demonstrated that herbivorous prey contains lower concentrations of insecticidal proteins than the plants they feed on and not all prey consumed by coccinellid species will have fed exclusively on DP51291 maize tissue. Coccinellid species are well known to be generalist feeders^87^ and have been noted to feed on their own eggs,^88–90^ the eggs of other Lepidoptera,^89^ and show some prey preference toward aphids^87^ and spider mite eggs.^57,61^ Aphids are phloem-feeders and have been noted to take up negligible concentrations of insecticidal proteins.^54–56^ In addition, exposure to concentrations of the IPD072Aa protein would likely be low in the spider mite eggs compared to the adult spider mites.^62^ Therefore, the assumption that 20% of the protein^62^ in the GM plant transfers to the coccinellid prey is likely quite conservative.

Degradation of the IPD072Aa protein in prey will further reduce exposure as the IPD072Aa protein will likely not persist in prey as insecticidal protein concentrations in prey have been noted to be lower than the plants they feed on.^54–56,59,60^ This is likely due to the nature of proteins and their ability to degrade by abiotic factors (i.e., photodegradation, pH, and temperature) and by biotic factors (i.e., microbial degradation).

Lastly, all MoE calculations were conducted based on the dry weight concentration of the IPD072Aa protein in DP51291 maize tissues. The dry weight concentrations are considered high estimates, since in reality NTOs would be exposed to levels comparable to fresh weight levels.^71^

#### Convergent Ladybird Beetle

Previously, *H. convergens* were exposed to the IPD072Aa protein and adverse effects on survival, weight, and days to emergence were reported at 1000 ng IPD072Aa protein/mg diet (survival was reduced by approximately 50% at this highest concentration).^8^ Further, at 500 ng IPD072Aa protein/mg diet, *H. convergens* also had significantly lower weight and a higher number of days to adult emergence. *H. convergens* exposure to 100 ng and 500 ng IPD072Aa protein/mg diet had no adverse effects on survival^8^ (Table 2). Therefore, the survival NOEC was determined to be 500 ng IPD072Aa protein/mg diet, and the corresponding MoE and refined MoEs for predators and parasitoids exposed to the IPD072Aa protein in DP51291 maize via prey are 3.57 and 36, respectively (Table 3). Based on the results of the hazard study and the refined EEC and MoE, no potential risk is anticipated as the NOEC is 36 times the refined EEC indicating a large margin of safety. As previously discussed for *C. maculata*, another coccinellid species, there are several factors will reduce the actual exposure to the IPD072Aa protein via the predator route below the worst-case EECs. These factors include: 1) food and prey choice; 2) degradation of the IPD072Aa protein in prey; and 3) fresh weight versus dry weight concentrations.

#### Parasitic Hymenoptera

*P. foveolatus* exposed to a diet containing a target concentration of 2000 µg IPD072Aa protein/ml demonstrated no adverse effects on survival (Supplemental Table 1). Therefore, the NOEC was determined to be 2000 µg IPD072Aa protein/ml diet,^8^ and the corresponding MoE is 14.3X the worst-case EEC for predators and parasitoids exposed to the IPD072Aa protein in DP51291 maize via prey (Table 3).

No biologically relevant adverse effects are expected on predators or parasitoids, including non-target Coccinellidae, due to cultivation of DP51291 maize. This conclusion for predators and parasitoids is consistent with the previous assessment for the IPD072Aa protein in the antecedent maize event, DP23211.^8^ Established guidance states that while Tier I testing typically targets a maximum hazard dose of ≥10X the EEC when possible, only observed effects at or below the EEC constitute possible environmental risk.^39^ Some statistical differences in lethal and sublethal endpoints were observed for *C. maculata* and *H. convergens* (Supplemental Table 1), however the magnitude of the effects and the resulting NOECs did not surpass established levels of concern for insecticidal proteins. Therefore, the results support the conclusion of minimal potential risk to predators and parasitoids, including non-target Coccinellidae, and higher-tier testing was not warranted.

### Insectivorous Birds

A previous laboratory study was conducted to assess the dietary effect of the IPD072Aa protein to a suitable surrogate species of insectivorous bird. *C. virginianus* were exposed to a nominal limit dose of 2000 mg IPD072Aa protein/kg body weight, and no mortality, abnormal behavior or signs of toxicity were observed (Table 2). Therefore, the LD_50_ was > 2000 mg IPD072Aa protein/kg body weight, the NOEC was 2000 mg IPD072Aa protein/kg body weight,^8^ and the corresponding MoE for wild birds that are exposed via an insectivorous route of exposure (predator) using the worst case EEC is 14.3 (Table 3). The overall MoE value indicates that potential risk to insectivorous birds is negligible which is further supported by the previous findings and assessment of the IPD072Aa protein in the antecedent maize event, DP23211.^8^ Therefore, the IPD072Aa protein in DP51291 maize is unlikely to be harmful to insectivorous birds at environmentally realistic concentrations.

### Granivorous Mammals (mouse)

A previous laboratory study was conducted to assess the dietary effect of the IPD072Aa protein to a suitable surrogate species of non-target granivorous mammals. *Mus musculus* were orally exposed at a dose of 2000 mg IPD072Aa protein/kg body weight and no mortality or other evidence of acute oral toxicity was observed based on evaluation of body weight, clinical signs, and gross pathology (Table 2). The LD_50_ for *M. musculus* was > 2000 mg IPD072Aa/kg body weight^8^ and corresponding MoE for wild mammals exposed via the grain feeding route of exposure is 691.8 (Table 3). The overall MoE value indicates that potential risk to granivorous mammals is negligible which is further supported by the previous findings and assessment of the IPD072Aa protein in the antecedent maize event, DP23211.^8^ Therefore, the IPD072Aa protein in DP51291 maize is unlikely to be harmful to granivorous mammals at environmentally realistic concentrations.

## Field Assessment of Non-Target Arthropods

The sites for the field assessment were selected in regions of maize cultivation such that collected taxa would be representative of maize ago-ecosystems. A total of 14 different species of NTAs, representing seven different functional groups, were identified via visual observation or collection (i.e., pitfall traps or sticky cards) across the three sites (i.e., Stewardson, IL, Atlantic, IA, and Germansville, PA; Table 4).

**Table 4.** Non-target Arthropods observed or collected during the DP51291 2022 field trials at Stewardson, IL; Atlantic, IA; or Germansville, PA.

Order	Family	Common Name	Taxa Preferred Name	Functional Group	Sampling Method
Hemiptera	Aphididae	Aphid	Aphididae	Herbivore	Visual observations
Hymenoptera	Apidae	Bee	Apidae	Pollen and Nectar User	Sticky card
Araneae		Spider	Araneae	Predator	Pitfall
					Visual observations
Hymenoptera	Braconidae	Parasitic Wasp	Braconidae	Parasitoid	Sticky card
Coleoptera	Carabidae	Ground Beetle	Carabidae	Predator	Pitfall
Neuroptera	Chrysopidae	Green Lacewing	*Chrysoperla* sp.	Omnivore	Sticky card
					Visual observations
Coleoptera	Coccinellidae	Ladybird Beetle	Coccinellidae	Omnivore	Sticky card
					Visual observations
Collembola		Springtail	Collembola	Detritivore	Pitfall
Hymenoptera	Ichneumonidae	Parasitic Wasp	Ichneumonidae	Parasitoid	Sticky card
Hemiptera	Nabidae	Damsel Bug	*Nabis* sp.	Predator	Sticky card
					Visual observations
Hemiptera	Anthocoridae	Minute pirate bug	*Orius insidiosus*	Predator	Sticky card
					Visual observations
Diptera	Syrphidae	Hover Fly	Syrphidae	Pollen and Nectar User	Sticky card
					Visual observations
Neuroptera	Hemerobiidae	Brown Lacewing	Hemerobiidae	Omnivore	Sticky card
					Visual observations
Hymenoptera	Megachilidae	Leafcutter Bee	Megachilidae	Pollen and Nectar User	Sticky card

From visual observations, the most abundant taxa were ladybird beetles (Coccinellidae) at Stewardson, IL, aphids (Aphididae) at Atlantic, IA, and minute pirate bug (*Orius insidiosus*) at Germansville, PA (Table 5). For taxa that met the minimum abundance criteria, no significant differences were observed between DP51291 and control maize for Aphididae at Atlantic, IA or *Nabis* sp and *Orius insidiosus* at Germansville, PA (Table 6). A linear mixed model analysis was used to analyze plot level diversity for the Shannon’s diversity index, Pielou’s evenness index, and Simpson’s diversity index. At all sites, no significant differences were observed between DP51291 maize and control maize in any of the three diversity indices suggesting similar diversity and evenness between DP51291 maize and control maize (Table 7).

**Table 5.** Non-target Arthropods observed with visual observations in DP51291 maize and control maize.

Taxa	DP51291 Maize		Control Maize		Total	
	Number	%	Number	%	Number	%
**Stewardson, IL**						
Aphididae	0	0.00	23	27.06	23	12.04
Araneae	27	25.47	31	36.47	58	30.37
*Chrysoperla* sp.	25	23.58	3	3.53	28	14.66
Coccinellidae	46	43.40	14	16.47	60	31.41
Hemerobiidae	0	0.00	2	2.35	2	1.05
*Nabis* sp.	0	0.00	1	1.18	1	0.52
*Orius insidiosus*	4	3.77	2	2.35	6	3.14
Syrphidae	4	3.77	9	10.59	13	6.81
**Total**	106	100	85	100	191	100
**Taxa Richness**	5	–	8	–	8	–
**Atlantic, IA**						
Aphididae	81	41.97	62	31.63	143	36.76
Araneae	27	13.99	33	16.84	60	15.42
Coccinellidae	35	18.13	43	21.94	78	20.05
*Orius insidiosus*	22	11.40	25	12.76	47	12.08
Syrphidae	28	14.51	33	16.84	61	15.68
**Total**	193	100	196	100	389	100
**Taxa Richness**	5	–	5	–	5	–
**Germansville, PA**						
Aphididae	11	2.76	7	1.83	18	2.30
Araneae	42	10.53	32	8.38	74	9.48
*Chrysoperla* sp.	3	0.75	8	2.09	11	1.41
Coccinellidae	39	9.77	37	9.69	76	9.73
*Nabis* sp.	54	13.53	42	10.99	96	12.29
*Orius insidiosus*	247	61.90	256	67.02	503	64.40
Syrphidae	3	0.75	0	0.00	3	0.38
**Total**	399	100	382	100	781	100
**Taxa Richness**	7	–	6	–	7	–

Note: Taxa richness is the number of taxa present.

**Table 6.** Across stage analysis for the non-target arthropod communities observed from visual observations in DP51291 maize and control maize.

Diversity Indices	Reported Statistics	DP51291 Maize	Control Maize
**Atlantic, IA**			
Aphididae	Mean	0.846	0.625
	95% Confidence Interval	0 - 2.82	0 - 2.36
	P-Value	0.2846	–
**Germansville, PA**			
*Nabis* sp	Mean	0.304	0.330
	95% Confidence Interval	0 - 0.753	0 - 0.788
	P-Value	0.8253	–
*Orius insidiosus*	Mean	1.05	1.14
	95% Confidence Interval	0.401 - 1.99	0.464 - 2.13
	P-Value	0.7846	–

**Table 7.** Linear mixed model results for plot level diversity across stage - ecological indices for the non-target arthropod communities observed from visual observations in DP51291 maize and control maize.

Diversity Indices	Reported Statistics	DP51291 Maize	Control Maize
**Stewardson, IL**			
Shannon’s Diversity Index	Mean	0.764	0.717
	95% Confidence Interval	0.394 - 1.13	0.346 - 1.09
	P-Value	0.7752	–
Pielou’s Evenness Index	Mean	0.890	0.905
	95% Confidence Interval	0.802 - 0.978	0.809 - 1.00
	P-Value	0.7823	–
Simpson’s Diversity Index	Mean	0.480	0.449
	95% Confidence Interval	0.243 - 0.716	0.214 - 0.684
	P-Value	0.7527	–
**Atlantic, IA**			
Shannon’s Diversity Index	Mean	0.898	0.963
	95% Confidence Interval	0.758 - 1.04	0.824 - 1.10
	P-Value	0.4124	–
Pielou’s Evenness Index	Mean	0.893	0.918
	95% Confidence Interval	0.809 - 0.977	0.833 - 1.00
	P-Value	0.4638	–
Simpson’s Diversity Index	Mean	0.524	0.572
	95% Confidence Interval	0.428 - 0.620	0.476 - 0.668
	P-Value	0.3393	–
**Germansville, PA**			
Shannon’s Diversity Index	Mean	0.868	0.811
	95% Confidence Interval	0.656 - 1.08	0.598 - 1.02
	P-Value	0.4430	–
Pielou’s Evenness Index	Mean	0.752	0.751
	95% Confidence Interval	0.647 - 0.856	0.647 - 0.856
	P-Value	0.9949	–
Simpson’s Diversity Index	Mean	0.488	0.474
	95% Confidence Interval	0.378 - 0.599	0.364 - 0.584
	P-Value	0.7254	–

NTAs collected from sticky traps are shown in Table 8. The most abundant taxa were parasitic wasps (Braconidae) at Stewardson, IL, and Atlantic, IA, and ladybird beetles (Coccinellidae) at Germansville, PA. For taxa that met the minimum abundance criteria, no significant differences were observed between DP51291 and control maize for Braconidae at Stewardship, IA, and Coccinellidae at Germansville, PA (Table 9). Like the results from the visual observations, a linear mixed model analysis was used to analyze plot level diversity across stage for the three diversity indices. At all sites, no significant differences were observed between DP51291 maize and control maize in any of the three diversity indices suggesting similar diversity and evenness between DP51291 maize and control maize (Table 10).

**Table 8.** Non-target Arthropods observed with sticky traps in DP51291 maize and control maize.

Taxa	DP51291 Maize		Control Maize		Total	
	Number	%	Number	%	Number	%
**Stewardson, IL**						
Apidae	1	0.71	1	0.68	2	0.69
Braconidae	80	56.74	93	62.84	173	59.86
*Chrysoperla* sp.	1	0.71	2	1.35	3	1.04
Coccinellidae	19	13.48	26	17.57	45	15.57
Hemerobiidae	6	4.26	5	3.38	11	3.81
Syrphidae	34	24.11	21	14.19	55	19.03
**Total**	141	100	148	100	289	100
**Taxa Richness**	6	–	6	–	6	–
**Atlantic, IA**						
Braconidae	91	75.21	77	68.75	168	72.10
*Chrysoperla* sp.	11	9.09	15	13.39	26	11.16
Coccinellidae	6	4.96	10	8.93	16	6.87
Hemerobiidae	3	2.48	1	0.89	4	1.72
Ichneumonidae	2	1.65	0	0.00	2	0.86
Syrphidae	8	6.61	9	8.04	17	7.30
**Total**	121	100	112	100	233	100
**Taxa Richness**	6	–	5	–	6	–
**Germansville, PA**						
Apidae	0	0.00	1	0.53	1	0.25
Braconidae	32	14.81	25	13.30	57	14.11
*Chrysoperla* sp.	4	1.85	3	1.60	7	1.73
Coccinellidae	175	81.02	151	80.32	326	80.69
Hemerobiidae	3	1.39	2	1.06	5	1.24
*Orius insidiosus*	0	0.00	2	1.06	2	0.50
Syrphidae	2	0.93	4	2.13	6	1.49
**Total**	216	100	188	100	404	100
**Taxa Richness**	5	–	7	–	7	–

Note: Taxa richness is the number of taxa present.

**Table 9.** Across stage analysis for the non-target arthropod communities observed from sticky traps in DP51291 maize and control maize.

Taxa	Reported Statistics	DP51291 Maize	Control Maize
**Stewardson, IL**			
Braconidae	Mean	3.49	4.48
	95% Confidence Interval	1.40 – 7.41	1.93 – 9.27
	P-Value	0.1595	–
**Germansville, PA**			
Coccinellidae	Mean	6.67	6.25
	95% Confidence Interval	4.19 – 10.3	3.91 – 9.71
	P-Value	0.4016	–

**Table 10.** Linear mixed model results for plot level diversity across stage - ecological indices for the non-target arthropod communities observed from sticky traps in DP51291 maize and control maize.

Diversity Indices	Reported Statistics	DP51291 Maize	Control Maize
**Stewardson, IL**			
Shannon’s Diversity Index	Mean	0.701	0.788
	95% Confidence Interval	0.551 - 0.850	0.638 - 0.937
	P-Value	0.3974	–
Pielou’s Evenness Index	Mean	0.809	0.818
	95% Confidence Interval	0.732 - 0.886	0.741 - 0.895
	P-Value	0.8607	–
Simpson’s Diversity Index	Mean	0.409	0.455
	95% Confidence Interval	0.336 - 0.482	0.382 - 0.529
	P-Value	0.3582	–
**Atlantic, IA**			
Shannon’s Diversity Index	Mean	0.474	0.593
	95% Confidence Interval	0.211 - 0.736	0.331 - 0.856
	P-Value	0.3613	–
Pielou’s Evenness Index	Mean	0.591	0.738
	95% Confidence Interval	0.464 - 0.718	0.627 - 0.850
	P-Value	0.0818	–
Simpson’s Diversity Index	Mean	0.261	0.352
	95% Confidence Interval	0.140 - 0.382	0.232 - 0.473
	P-Value	0.2113	–
**Germansville, PA**			
Shannon’s Diversity Index	Mean	0.554	0.589
	95% Confidence Interval	0.339 - 0.770	0.373 - 0.804
	P-Value	0.7437	–
Pielou’s Evenness Index	Mean	0.617	0.610
	95% Confidence Interval	0.497 - 0.737	0.490 - 0.731
	P-Value	0.8982	–
Simpson’s Diversity Index	Mean	0.326	0.329
	95% Confidence Interval	0.199 - 0.453	0.202 - 0.456
	P-Value	0.9609	–

The NTAs collected from pitfall traps are shown in Table 11. The most abundant taxa were spiders (Araneae) and ground beetles (Carabidae) at Stewardson, IL, and springtails (Collembola) at Atlantic, IA, and Germansville, PA. At all sites, taxa richness was the same in DP51291 maize and control maize. For taxa that met the minimum abundance criteria, no significant differences were observed between DP51291 and control maize for Araneae, Carabidae, or Collembola, in addition, no differences were observed for Araneae or Collembola at Germansville, PA (Table 12). Based on a linear mixed model analysis to analyze plot level diversity across stage for the three diversity indices, no significant differences were observed between DP51291 maize and control maize in any of the three diversity indices. This suggests a similar diversity and evenness between DP51291 maize and control maize (Table 13).

**Table 11.** Non-target Arthropods observed with pitfall traps in DP51291 maize and control maize.

Taxa	DP51291 Maize		Control Maize		Total	
	Number	%	Number	%	Number	%
**Stewardson, IL**						
Araneae	628	34.93	450	26.77	1078	30.99
Carabidae	606	33.70	720	42.83	1326	38.11
Collembola	564	31.37	511	30.40	1075	30.90
**Total**	1798	100.00	1681	100.00	3479	100.00
**Taxa Richness**	3	–	3	–	3	–
**Atlantic, IA**						
Araneae	162	8.37	181	9.09	343	8.73
Carabidae	333	17.21	306	15.36	639	16.27
Collembola	1440	74.42	1505	75.55	2945	74.99
**Total**	1935	100.00	1992	100.00	3927	100.00
**Taxa Richness**	3	–	3	–	3	–
**Germansville, PA**						
Araneae	149	32.18	125	30.86	274	31.57
Carabidae	54	11.66	44	10.86	98	11.29
Collembola	260	56.16	236	58.27	496	57.14
**Total**	463	100.00	405	100.00	868	100.00
**Taxa Richness**	3	–	3	–	3	–

Note: Taxa richness is the number of taxa present.

**Table 12.** Across stage analysis for the non-target arthropod communities observed from pitfall traps in DP51291 maize and control maize.

Diversity Indices	Reported Statistics		DP51291 Maize		Control Maize	
**Stewardson, IL**						
Araneae		Mean		11.6		10.2
		95% Confidence Interval		6.65 - 19.6		5.84 - 17.5
		P-Value		0.5214		–
Carabidae		Mean		11.1		13.7
		95% Confidence Interval		8.11 - 15.1		10.0 - 18.5
		P-Value		0.1152		–
Collembola		Mean		13.5		13.2
		95% Confidence Interval		10.4 - 17.5		10.2 - 17.1
		P-Value		0.8886		–
**Atlantic, IA**						
Araneae		Mean		4.17		5.05
		95% Confidence Interval		2.59 - 6.44		3.20 - 7.71
		P-Value		0.3657		–
Carabidae		Mean		8.44		7.56
		95% Confidence Interval		4.50 - 15.2		3.99 - 13.7
		P-Value		0.5126		–
Collembola		Mean		22.1		23.6
		95% Confidence Interval		12.9 - 37.2		13.8 - 39.7
		P-Value		0.7798		–
**Germansville, PA**						
Araneae		Mean		5.49		5.04
		95% Confidence Interval		2.95 - 9.66		2.67 - 8.92
		P-Value		0.7507		–
Collembola		Mean		6.75		6.30
		95% Confidence Interval		5.60 - 8.10		5.21 - 7.57
		P-Value		0.4993		–

**Table 13.** Linear mixed model results for plot level diversity across stage - ecological indices for the non-target arthropod communities observed from pitfall traps in DP51291 maize and control maize.

Diversity Indices	Reported Statistics	DP51291 Maize	Control Maize
**Stewardson, IL**			
Shannon’s Diversity Index	Mean	0.923	0.954
	95% Confidence Interval	0.887 - 0.959	0.918 - 0.990
	P-Value	0.2122	–
Pielou’s Evenness Index	Mean	0.840	0.868
	95% Confidence Interval	0.808 - 0.873	0.836 - 0.901
	P-Value	0.2122	–
Simpson’s Diversity Index	Mean	0.549	0.569
	95% Confidence Interval	0.524 - 0.574	0.544 - 0.594
	P-Value	0.2537	–
**Atlantic, IA**			
Shannon’s Diversity Index	Mean	0.793	0.783
	95% Confidence Interval	0.720 - 0.866	0.710 - 0.855
	P-Value	0.8359	–
Pielou’s Evenness Index	Mean	0.722	0.712
	95% Confidence Interval	0.655 - 0.788	0.646 - 0.779
	P-Value	0.8359	–
Simpson’s Diversity Index	Mean	0.460	0.454
	95% Confidence Interval	0.413 - 0.507	0.408 - 0.501
	P-Value	0.8637	–
**Germansville, PA**			
Shannon’s Diversity Index	Mean	0.837	0.806
	95% Confidence Interval	0.715 - 0.958	0.685 - 0.928
	P-Value	0.6140	–
Pielou’s Evenness Index	Mean	0.761	0.743
	95% Confidence Interval	0.662 - 0.861	0.643 - 0.842
	P-Value	0.7031	–
Simpson’s Diversity Index	Mean	0.507	0.480
	95% Confidence Interval	0.430 - 0.583	0.404 - 0.556
	P-Value	0.4716	–

Overall, the results of the supporting field assessment indicate that DP51291 maize did not have an effect on the abundance, evenness, or diversity of NTAs when compared to the control maize. Field studies are typically conducted only when triggered by the tiered testing strategy and risk assessment framework. In this case, the results of the Tier I laboratory hazard bioassays and risk characterization of the IPD072Aa protein indicated minimal risk to NTOs from the cultivation of DP51291 maize, eliminating the need for higher-tier testing. This conclusion is further supported by the field assessment that corroborated the results from conservative Tier 1 laboratory hazard bioassays and exposure assumptions used in the risk characterization. While providing additional confidence to the risk assessment, the field study provided also demonstrates that conservative Tier I testing was sufficient to make conclusions of minimal risk to NTOs for DP51291 maize. These data further add to the body of evidence that the biodiversity of NTAs are not impacted due to GM crops.^91,92^

## Conclusions

The previously generated data, risk assessment,^8^ and regulatory decisions for IPD072Aa by the US EPA^29^ and USDA^30,31^ for the antecedent maize event DP23211 were used to inform and support the problem formulation and risk assessment for the maize event DP51291. Reasonable worst-case EECs of the IPD072Aa protein in DP51291 maize were determined using conservative assumptions to determine potential exposure of NTOs. Several factors that reflect more realistic environmental conditions were used to refine EECs when needed to determine the likelihood of unreasonable adverse effects to NTOs. Utilizing the robust and well-established framework set forth by USDA and US EPA, the results of the risk assessment support the conclusion that no unreasonable adverse effects to NTO populations are anticipated from the cultivation of DP51291 maize. This assessment serves as a strong example of how prior risk assessment can inform problem formulation and streamline NTO testing of insecticidal proteins expressed in GM crops.

## Supplementary Material

Supplemental Material
