# Supplementary material for: An environmental risk assessment of maize containing event, DP-Ø51291–2, with activity against corn rootworms (Diabrotica spp.) via expression of the protein, IPD072Aa
Source: GM Crops Food. 2025 Oct 22;16(1):811–36. doi: 10.1080/21645698.2025.2572192 (PMC12548076; doi:10.1080/21645698.2025.2572192)
Supplement: Supplemental Material [file KGMC_A_2572192_SM8421.docx]

**Supplementary Table 1:** Tier I Hazard Laboratory Bioassay Results for the IPD072Aa protein in DP51291 Maize on Representative Surrogate Species.

| **Functional Group** | **Species  (Common Name)** | **Concentration(s) tested** | **Mortality (%)**  **(*P*-value*)*** | **Sublethal Endpoint A**  **(*P-*value)** | **Sublethal Endpoint B**  **(*P-*value)^c^** |
| --- | --- | --- | --- | --- | --- |
| ***Aquatic Organisms*** | *Daphnia magna* ^a^  (water flea) | 0 mg IPD072Aa/L | 0% | 127 | NA |
|  |  | 15 mg IPD072Aa /L | 0% (1.000) | 126  (0.4602) | NA |
| ***Soil Dwelling Decomposers & Detritivores*** | *Folsomia candida* ^b^ (springtail) | 0 ng IPD072Aa/mg diet | 5.00% | 515 | NA |
|  |  | 4000 ng IPD072Aa/mg diet | 6.25% (0.500) | 532 (0.6340) | NA |
|  |  | 1000 ng teflubenzuron/mg diet ^†^ | 100% | 0 | NA |
| ***Predators & Parasitoids*** | *Chrysoperla* ^c^ *rufilabris*  (green lacewing) | 0 ng IPD072Aa/mg diet | 10% | 91.7% | 12.9 |
|  |  | 2000 ng IPD072Aa/mg diet | 17.5% (0.2589) | 100% (1.000) | 15.8 (<0.0001)**^*^** |
|  |  | 25000 ng cryolite/mg diet ^†^ | 89.7% | 25% | 21.0 |
|  | *Pediobius* ^d^ *foveolatus*  (parasitic hymenoptera) | 0 µg IPD072Aa/ml diet | 13.8% | NA | NA |
|  |  | 2000 µg IPD072Aa/ml diet | 13.8% (0.6471) | NA | NA |
|  |  | 20000 µg boric acid/ml diet ^†^ | 96.7% | NA | NA |

^a^ Daphnia sublethal endpoint A was reproduction (i.e., mean number of offspring per surviving daphnid). N = 10 organisms per treatment at test initiation.

^b^ Springtail, the sublethal endpoint A was reproduction (i.e., mean number of offspring per jar). N = 80 organisms per treatment at test initiation.

^c^ Green lacewing sublethal endpoint A was pupation rate (%), and sublethal endpoint B was the mean number of days to pupation. N = 40 larvae per treatment at test initiation. A significant increase in days to pupation was observed compared to the control (0 ng IPD072Aa protein/mg diet). The delay in pupation represents less than a 50% effect relative to the control and is not considered biologically meaningful as the typical trigger to advance to a tier 2 study is set to a 50% effect level (Rose, 2007).

^d^ *Pediobius faveolatus* had no sublethal endpoints assessed. N = 30 organisms per treatment at test initiation.

NA: Not applicable as no additional sublethal endpoint was assessed.
^†^Positive control for bioassay

* A statistically significant difference (*P*-value < 0.05) was observed.
